# Correction: Laws for health and care worker protection and rights: A study of 182 countries

**DOI:** 10.1371/journal.pgph.0005007

**Published:** 2025-07-31

**Authors:** Matthew M. Kavanagh, Adi Radhakrishnan, Vishakh Unnikrishnan, Giorgio Cometto, Catherine Kane, Eric A. Friedman, Varsha Srivatsan, Luis Gil Abinader, James Campbell, on behalf of the Health & Care Worker Policy Lab

The second author’s name is spelled incorrectly. The correct name is: Adi Radhakrishnan. The correct citation is: Kavanagh MM, Radhakrishnan A, Unnikrishnan V, Cometto G, Kane C, Friedman EA, et al. (2024) Laws for health and care worker protection and rights: A study of 182 countries. PLOS Glob Public Health 4(12): e0003767. https://doi.org/10.1371/journal.pgph.0003767.
