# Supplementary material for: Laws for health and care worker protection and rights: A study of 182 countries
Source: PLOS Glob Public Health. 2024 Dec 9;4(12):e0003767. doi: 10.1371/journal.pgph.0003767 (PMC11627435; doi:10.1371/journal.pgph.0003767)
Supplement: S1 Text — (DOCX) [file pgph.0003767.s001.docx]

***Supporting Information:***

**S 1: Health & Care Worker Compact Team***

*authorship based on ICME definition.

Center for Global Health Policy & Politics, O’Neill Institute for National and Global Health Law, Georgetown University

- Matthew M. Kavanagh, PhD, Director
- Varsha Srivatsan, MA, Program Manager
- Adi Radakrishnan, JD, Law Fellow
- Vishakh Unnikrishnan, MPH, Associate
- Eric A. Friedman, JD, Global Health Justice Fellow
- Luis Gil Abinader, LLM, Law Fellow

Research Assistants

- Ayush Sharma
- Bakary Fatty
- Claudia Lorena Díaz
- Demoz Hamda
- Laura Juvé Sánchez
- Tommy Dell
- Hyland Brown
- Zarif Hosain

World Health Organization Department of Health Workforce

- James Campbell, PhD, Director
- Giorgio Cometto, MSc, Coordinator, Human resources for health policies, norms and standards
- Catherine Kane, MCD, CEM, Technical Officer

Normative, Codebook and Data Reviewers

- Howard Catton, International Council of Nurses
- Hoi Shan Fokeladeh, International Council of Nurses
- Dr Catherine Duggan, FIP, International Pharmaceutical Federation
- Dr Otmar Kloiber, World Medical Association
- Dr. Julia Tainijoki-Seyer, World Medical Association
- Dr Caline Mattar, Global Health Workforce Network Youth Hub
- Baba Aiyelabola, Public Service International
- NR Dr Stefano Scarpetta, Organisation for Economic Co-operation and Development
- Maren Hopfe, International Labor Organization
- NR Leonard Rubenstein, Safeguarding Health in Conflict Coalition
- NR Tina Flores, Frontline Health Worker Coalition
- Dr Ivan Ivanov, WHO Occupational and Workplace Health
- Dorothy Ngajilo, WHO Occupational and Workplace Health
- NR Hyo Jeong Kim, WHO Health Emergencies
