# Supplementary material for: Laws for health and care worker protection and rights: A study of 182 countries
Source: PLOS Glob Public Health. 2024 Dec 9;4(12):e0003767. doi: 10.1371/journal.pgph.0003767 (PMC11627435; doi:10.1371/journal.pgph.0003767)
Supplement: S2 Text — (DOCX) [file pgph.0003767.s002.docx]

***Supporting Information:***

**S 2: Methodology**

**Data Sources**

**National Law and Policy Documents**

Primary law and policy documents (including legislation, regulations, directives, circulars, etc.) were collected from online legal databases, national government or parliamentary websites, existing labor or health law repositories, and through inquiries to national experts.

The Health & Care Worker Policy Lab includes materials on the Global Health and Care Worker Compact including a full library with all the primary legal documents used in this paper. It can be found at [www.HCWPolicyLab.org](http://www.HCWPolicyLab.org).

**World Health Organization National Health Workforce Accounts (NHWA)**

The WHO’s NHWA is a central source of information for this paper and where possible we have aligned indicators with that source to facilitate sustainable data collection. The purpose of the NHWA is to facilitate the standardization of human resources for health information systems for interoperability, i.e. the ability to exchange health workforce (HWF) data within broader subnational or national health information systems (HIS), as well as within international databases. Since the launch of the NHWA in 2017, there has been a tremendous improvement in global HWF data availability and quality. The NHWA has succeeded in its mandate of fostering a harmonized approach for the annual and timely collection of HWF information and defining core indicators in support of strategic workforce planning and global monitoring. As of April 2023, 181 countries, territories, and areas have appointed national focal points for annual reporting of HWF data on the NHWA data platform. Meanwhile, the NHWA data portal contains the latest available global HWF data for key indicators, country profiles, occupation profiles and other visualizations, and boasts more than 4,800 registered users.^1^ The NHWA in its current form has modules on stock and flow that provide a comprehensive overview of the composition and distribution of the HWF, and their participation in the health labor market; Education, which provides information on HWF education and training capacity, applications, enrolments, and graduations; Finance and expenditure which maps expenditures on the HWF and remunerations in the health sector; and Working conditions, governance and leadership, which includes indicators on working conditions, HWF governance and leadership capacity, and on the capacity of national HRHIS.

**Table A**

From the NWHW we made use of the following indicators:

| Compact Indicator | NHWA Indicator |
| --- | --- |
| 2 | 4-01.7 Existence of national/subnational care packages for mental well being of health workers (Yes/Partial/No) |
| 3 | 4-01.6 Existence of national/subnational policies/laws for prevention of attacks on health workers (Yes/Partial/No) |
| 6 | 4-01.1 Existence of national/subnational policies/laws regulating working hours and conditions (Yes/Partial/No)  4-01.2 Existence of national/subnational policies/laws regulating minimum wage (Yes/Partial/No) |
| 7 | 4-01.3 Existence of national/subnational policies/laws regulating social protection (Yes/Partial/No) |
| 8 | 2-07.5 Existence of national systems for continuing professional development (Yes/Partial/No)  2-07.6 Existence of in-service training as an element of national education plans for the health workforce (Yes/Partial/No) |

**Global Health Observatory Database (GHO)**

The GHO data repository is WHO's gateway to health-related statistics for its 194 Member States. It provides access to over 1000 indicators on priority health topics including mortality and burden of diseases, the Millennium Development Goals (child nutrition, child health, maternal and reproductive health, immunization, HIV/AIDS, tuberculosis, malaria, neglected diseases, water and sanitation), non communicable diseases and risk factors, epidemic-prone diseases, health systems, environmental health, violence and injuries, equity among others.

We made use of the following indicator:

| Compact Indicator | WHO Indicator |
| --- | --- |
| 1 | Existence of national policy instruments for occupational health and safety for health workers |

**International Labour Organization Global Care Policy Portal**

The ILO Global Care Policy portal is a knowledge hub to disseminate data and resources on care leave policies and services to advance the ILO transformative agenda for gender equality and non-discrimination. The portal presents over 60 legal and statistical indicators on maternity protection, paternity leave, parental leave and other care leave and non-discrimination policies, as well as childcare and long-term care services in more than 180 countries. It uses international labour standards as benchmarks and builds on ILO policy research since 1994.

We made use of the following indicator:

| Compact Indicator | ILO Indicator |
| --- | --- |
| 7 | Duration of parental leave available to households (weeks) |
