# Supplementary material for: Laws for health and care worker protection and rights: A study of 182 countries
Source: PLOS Glob Public Health. 2024 Dec 9;4(12):e0003767. doi: 10.1371/journal.pgph.0003767 (PMC11627435; doi:10.1371/journal.pgph.0003767)
Supplement: S3 Text — (DOCX) [file pgph.0003767.s003.docx]

***Supporting Information:***

**S 3: Data supplement**

*Table A: Data from Figure 3*

|  | Yes | Partial | No | Coverage (% of countries with data) |
| --- | --- | --- | --- | --- |
| 1. Occupational Health | 107 | 28 | 18 | 84% |
| 2. Health Services | 27 | 94 | 16 | 75% |
| 3. Violence and Harassment | 86 | NA | 77 | 90% |
| 4. Conflict Protection | 71 | 20 | 22 | 62% |
| 5. Non-Discrimination | 20 | 57 | 51 | 70% |
| 6. Fair Remuneration | 160 | 11 | 1 | 95% |
| 7. Social Protection | 46 | 5 | 102 | 84% |
| 8. Work Environments | 139 | 13 | 23 | 96% |
| 9. Collective Bargaining | 111 | 40 | 4 | 85% |
| 10. Whistleblower Protections | 52 | 20 | 43 | 63% |

*Table B: Alignment Ratio by Income Level:*

| ***Income Level*** | ***Percentage of Ys*** |
| --- | --- |
| *Upper Middle Income* | *63%* |
| *Lower Middle Income* | *54%* |
| *Low* | *47%* |
| *High* | *74%* |

*Testing for income level as a significant predictor for indicator alignment:*

*Table C:* Analysis of Variance Results:

| **Source** | **Df** | **Sum Sq** | **Mean Sq** | **F value** | **Pr(>F)** |
| --- | --- | --- | --- | --- | --- |
| Income Level | 3 | 0.862 | 0.28740 | 9.562 | 0.00000691 |
| Residuals | 178 | 5.350 | 0.03006 |  |  |

*Table* D: Pairwise Results using Tukey-Kramer test:

| **Contrast** | **Estimate** | **SE** | **Df** | **t.ratio** | **p.value** |
| --- | --- | --- | --- | --- | --- |
| High - Low | 0.1879 | 0.0423 | 178 | 4.441 | 0.0001 |
| High - Lower Middle Income | 0.1537 | 0.0349 | 178 | 4.401 | 0.0001 |
| High - Upper Middle Income | 0.0887 | 0.0325 | 178 | 2.730 | 0.0349 |
| Low - Lower Middle Income | -0.0341 | 0.0440 | 178 | -0.776 | 0.8654 |
| Low - Upper Middle Income | -0.0992 | 0.0421 | 178 | -2.357 | 0.0893 |
| Lower Middle Income - Upper Middle Income | -0.0651 | 0.0347 | 178 | -1.877 | 0.2416 |

**Conclusion:** Significant differences in mean alignment values found between High income countries and countries classified as Low and Lower Middle Income (p-value<0.01). No significant differences between other income categories.

*Table* E: *Alignment across WHO Regions:*

| **Region** | **Percentage of Ys** |
| --- | --- |
| African Region (AFR) | 55% |
| Eastern Mediterranean Region (EMR) | 56% |
| European Region (EUR) | 79% |
| Region of the Americas (AMR) | 56% |
| South-East Asian Region (SEAR) | 55% |
| Western Pacific Region (WPR) | 58% |

*Table* F: *Testing for WHO region as a significant predictor for indicator alignment:*

| **Source** | **Df** | **Sum** **Sq** | **Mean Sq** | **F value** | **Pr(>F)** |
| --- | --- | --- | --- | --- | --- |
| WHO Region | 5 | 0.970 | 0.19400 | 6.513 | 0.0000139 |
| Residuals | 176 | 5.242 | 0.02979 |  |  |

**Conclusion:** Significant difference found between mean alignment values across WHO regions (p<0.05).

*Table* G: Pairwise Results using Tukey-Kramer test:

| **Contrast** | **Estimate** | **SE** | **Df** | **t.ratio** | **p.value** |
| --- | --- | --- | --- | --- | --- |
| African Region (AFR) - Eastern Mediterranean Region (EMR) | 0.02746 | 0.0494 | 176 | 0.555 | 0.9937 |
| African Region (AFR) - European Region (EUR) | -0.15193 | 0.0356 | 176 | -4.271 | 0.0005 |
| African Region (AFR) - Region of the Americas (AMR) | -0.00477 | 0.0396 | 176 | -0.120 | 1.0000 |
| African Region (AFR) - South-East Asian Region (SEAR) | 0.05267 | 0.0606 | 176 | 0.869 | 0.9533 |
| African Region (AFR) - Western Pacific Region (WPR) | 0.00144 | 0.0429 | 176 | 0.034 | 1.0000 |
| Eastern Mediterranean Region (EMR) - European Region (EUR) | -0.17939 | 0.0482 | 176 | -3.720 | 0.0036 |
| Eastern Mediterranean Region (EMR) - Region of the Americas (AMR) | -0.03223 | 0.0513 | 176 | -0.629 | 0.9888 |
| Eastern Mediterranean Region (EMR) - South-East Asian Region (SEAR) | 0.02521 | 0.0688 | 176 | 0.367 | 0.9991 |
| Eastern Mediterranean Region (EMR) - Western Pacific Region (WPR) | -0.02602 | 0.0538 | 176 | -0.483 | 0.9967 |
| European Region (EUR) - Region of the Americas (AMR) | 0.14716 | 0.0381 | 176 | 3.866 | 0.0021 |
| European Region (EUR) - South-East Asian Region (SEAR) | 0.20460 | 0.0596 | 176 | 3.433 | 0.0095 |
| European Region (EUR) - Western Pacific Region (WPR) | 0.15337 | 0.0415 | 176 | 3.700 | 0.0039 |
| Region of the Americas (AMR) - South-East Asian Region (SEAR) | 0.05744 | 0.0621 | 176 | 0.925 | 0.9396 |
| Region of the Americas (AMR) - Western Pacific Region (WPR) | 0.00621 | 0.0450 | 176 | 0.138 | 1.0000 |
| South-East Asian Region (SEAR) - Western Pacific Region (WPR) | -0.05123 | 0.0642 | 176 | -0.798 | 0.9676 |

*P value adjustment: tukey method for comparing a family of 6 estimates*

**Conclusion:** Significant differences in mean alignment values were observed between the European Region and the African Region (AFR), Eastern Mediterranean Region (EMR), Region of the Americas, South-East Asian Region and Western Pacific Region (WPR) (p < 0.01). In each case, the European Region showed significantly higher mean values compared to these regions. No significant differences found between mean alignment values across other regions.

**References**

1. WHO. National health workforce accounts: a handbook [Internet]. Geneva: World Health Organization; 2023 [cited 2024 Mar 23]. Available from: https://iris.who.int/bitstream/handle/10665/374320/9789240081291-eng.pdf?sequence=1
