# Supplementary material for: Laws for health and care worker protection and rights: A study of 182 countries
Source: PLOS Glob Public Health. 2024 Dec 9;4(12):e0003767. doi: 10.1371/journal.pgph.0003767 (PMC11627435; doi:10.1371/journal.pgph.0003767)
Supplement: S4 Text — (DOCX) [file pgph.0003767.s004.docx]

***Supporting Information:***

**S 4: Codebook**

## I.1 Protection from Occupational Hazards

- Question
  - Does the country have national policy instruments/laws for occupational health and safety that cover all health workers and require all health workers to have access to protective equipment or supplies?
- Coding Rule for Primary Source
  - Yes = National policy or law on occupational health and safety exists that covers all health workers, and explicitly obligates employers to either provide protective equipment or supplies to all health workers (e.g., PPE) or ensure that all health workers have access to protective equipment or supplies (from the government or their employer) in the workplace.
  - Partial = National law or policy on occupational health and safety exists that covers all health workers but does not obligate employers to provide protective equipment or supplies to health workers
  - No = A national policy or law either does not exist at all or it exists, but it does not cover all health workers.

- - ND = Research did not yield any law or policy for occupational health and safety that looks at protective equipment and supplies, i.e., such a policy could exist but we couldn’t find it.
- Benchmark
  - The health and safety of health and care workers should be protected within an overall approach of prevention, risk assessment, and implementation of control measures, and broader occupational safety and health principles and frameworks. Compact at para. 26.
- Hierarchy
  - N/A

## I.2 Health Services for Health and Care Workers

- Question
  - Does the country have policies/laws that allow for a national package of health services for health workers that includes mental well-being?
- Coding Rule for Primary Source
  - Yes = National policy/law establishes that employees are eligible to receive free medical treatment for occupational diseases or injuries including mental health, and the national health service package covers all health workers.

This may be available in regulations or directives, national health service plans, or national occupational health plans. The coverage scheme may either require employers to cover health services for occupational diseases or injuries directly, or indirectly by requiring employers to pay into a fund (e.g., an occupational injury scheme that is then managed by a central authority). All health workers should then be able to access services by receiving services via this occupational health fund free of charge.

- - - To be fully considered as “yes,” the diseases/injuries covered by any health service scheme should include services for mental health conditions and well-being. In other words, services for mental health conditions should be included in any definition or other explanation of what health services or conditions are included in defining “covered” diseases or injuries.
    - Per the WHO definition, mental health conditions include “mental disorders and psychosocial disabilities as well as other mental states associated with significant distress, impairment in functioning, or risk of self-harm. People with mental health conditions are more likely to experience lower levels of mental well-being, but this is not always or necessarily the case.”
  - Partial = There exists a policy/law that establishes coverage for occupational disease and injury that applies to all health workers BUT does not include mental health conditions and well-being.
  - No = There does not exist any national policy or law that includes free coverage for occupational disease and injury for all health workers.
  - ND = Research did not yield any law or policy, i.e., such a policy could exist but could not be found.
- Coding Rule for NHWA Source
  - *(N/A for Phase 1 – there is no corresponding NHWA 1.0 data set for this question.)*
- Benchmark
  - Quality, accessible, affordable, acceptable, and confidential health care must be available for all health and care workers. Free services and support should be available for diseases, injuries, and other conditions resulting from their work. Compact at para. 27.
- Hierarchy
  - N/A *for Phase 1*

## I.3 Protection Against Violence and Harassment

- Question
  - Does the country have national policies/laws for the prevention of attacks against all health workers?
- Coding Rule for Primary Source
  - Yes = A national policy/law includes at least one of the following measures that are aimed at reducing violence against, and harassment of, all health workers.
    - Heightened penalties for violence against health and care workers
    - Requirements on reporting violence against health and care workers
    - Mandatory training on workplace harassment
    - Health workplace security measures
    - Explicit protections for care workers against any immigration enforcement consequences if they report workplace violence,
    - The right of health and care workers to remove themselves from situations where they reasonably believe there is a serious and imminent risk of violence or harassment against them.
  - No = No policy/law exists that includes any of the measures enumerated above that are specifically aimed at reducing violence against, and harassment of, all health workers.

- - ND = Research did not yield any law or policy, i.e., such a policy could exist but could not be found.
- Coding Rule for NHWA Source
  - Yes = If the most recent response on NHWA 1.0 6-09 is “yes” or “partial”
  - No = If the most recent response on NHWA 1.0 6-09 is “no”
- Benchmark
  - All health and care workers should be protected from violence, sexual abuse and exploitation, and harassment in the world of work. Compact at para. 28.
- Hierarchy
  - If there is a conflict between the coding of a primary source, and the results from the NHWA, highlight the conflict. Results from NHWA are voluntarily reported by governments and are not validated by any external source. In most situations, the conclusions drawn from our primary source analysis will be superior to NHWA data. The final decision will be made upon team review.

## I.4 Protection Against Attacks in Fragility, Conflict, and Violence

- Question
  - Does the country have laws and policies that incorporate all Geneva Convention protections of health and humanitarian workers, health facilities, transport, and patients during armed conflict into national law?
- Coding Rule for Primary Source
  - Yes = Legal stipulations exist that incorporate the full extent of the Geneva Convention protections of health workers and infrastructure into domestic policy or law. This includes at least one of the following:
    - Protections against killing and violence of protected persons and attacks on protected facilities
    - Domestic criminal penalties for committing a war crime in violation of the Geneva Conventions.
  - Partial = Legal stipulations exist, but do not incorporate the full extent of the Geneva Convention protections of health workers and infrastructure.
    - *(For example, domestic law criminalizes killing protected persons but does not include violence or attacks against protected persons as a part of the crime.)*
  - No = There are no legal stipulations that incorporate the Geneva Convention protections of health workers and infrastructure into domestic policy or law.
  - ND = Research did not yield any law or policy, i.e., such a policy could exist but could not be found.
- Benchmark
  - In situations of fragility, conflict, and violence, it is imperative that all States Parties fulfill IHL obligations with respect to health and care work. Compact at para. 29.
- Hierarchy
  - N/A

## I.5 Equal Treatment and Non-Discrimination

- Question
  - Does the country have a law that prohibits discrimination in the workplace, on internationally protected grounds, that includes all health and care workers?
- Coding Rule for Primary Source
  - Yes = The country has a policy or law that prohibits discrimination in the workplace and also covers all internationally protected grounds, and either explicitly includes health and care workers or does not explicitly exclude them.
    - Internationally protected classes include “race, colour, sex, language, religion, political or other opinion, national or social origin, property, birth, … disability, age, nationality, marital and family status, sexual orientation or gender identity, health status, place of residence, and economic/social situation” per paragraphs 18-35 of General Comment 20 of CESCR
      - General Comment 20 makes it clear that the coverage of non-discrimination on the basis of nationality does not make distinctions given legal, immigration, or documentation status.
    - A general equal protection clause in a constitutional provision is not sufficient for this indicator to be marked as yes.
  - Partial = The country has a policy or law that protects both health and care workers from discrimination in the workplace (or does not explicitly exclude them) and covers at least one, but not all, of the internationally protected grounds.
  - No = The country lacks a policy or law that protects health and care workers from discrimination in the workplace (whether because the law does not exist at all or explicitly excludes health and care workers).
  - ND = Research did not yield any law or policy, i.e., such a policy could exist but could not be found.
- Benchmark
  - All health and care workers merit equal treatment and should be protected against discrimination on all internationally protected grounds, including the facilitation of women’s economic inclusion in the health and care economy. Compact at para. 30.
- Hierarchy
  - N/A

## I.6 Fair and Equitable Remuneration

- Question
  - Does the country have national policies/laws covering health workers that: 1) regulate working hours and conditions, and 2) regulate a minimum wage?
- Coding Rule for Primary Source
  - Yes = The country has a national policy/law that regulates both working hours and conditions, and a minimum wage, for health workers
  - Partial = The country has a national policy/law that regulates either working hours and conditions, or a minimum wage, but not both, for health workers
  - No = The country lacks a national policy/law that regulates working hours and conditions and a minimum wage, for health workers.
  - ND = Research did not yield any law or policy, i.e., such a policy could exist but could not be found.
  - *Sub-Indicators*
    - Sub i: Does the country have national policies/laws covering health workers that regulate *working hours and conditions*?
      - Yes = The country has a policy or law that ensures health workers are fairly remunerated for all hours worked, including overtime provisions. This includes regulations that set a maximum number of working days per week, a premium for night work, and work on a weekly rest day or holiday work.
      - No = The country lacks a policy or law that ensures the working hours and conditions of work are regulated for health workers.
      - ND = Research did not yield any law or policy, i.e., such a policy could exist but could not be found.
    - Sub ii: Does the country have national policies/laws covering health workers that regulate *a minimum wage*?
      - Yes = Health workers are eligible to receive a minimum wage according to national policies/laws.
      - No = Health workers are not eligible to receive a minimum wage according to national policies/laws.
      - ND = Research did not yield any law or policy, i.e., such a policy could exist but could not be found.
- Coding Rule for NHWA Source
  - Yes = If both sub-indicators are yes
  - Partial = If one sub-indicator is yes, but the other is no.
  - No = If both sub-indicators are no
  - *Sub-Indicators*
    - Sub i: Does the country have national policies/laws covering health workers that regulate *working hours and conditions*?
      - Yes = If the most recent response on NHWA 1.0 6-03 is “yes” or “partial”
      - No = If the most recent response on NHWA 1.0 6-03 is “no”
    - Sub ii: Does the country have national policies/laws covering health workers that regulate *a minimum wage*?
      - Yes = If the most recent response on NHWA 1.0 6-04 is “yes” or “partial”
      - No = If the most recent response on NHWA 1.0 6-04 is “no”
- Benchmark
  - It is important to ensure just and fair remuneration for all health and care workers, including community health, at levels comparable to other workers and professionals of similar/equivalent qualifications, responsibilities, workload, and risk with consideration to the cost of living. Compact at para. 31.
- Hierarchy
  - If there is a conflict between the coding of a primary source, and the results from the NHWA, highlight the conflict. Results from NHWA are voluntarily reported by governments and are not validated by any external source. In most situations, the conclusions drawn from our primary source analysis will be superior to NHWA data. The final decision will be made upon team review.

## I.7 Social Protection

- Question
  - Does the country have national social protection policies/laws that, at minimum, provide parental leave to all health workers?
- Coding Rule for Primary Source
  - Yes = The country has social protection measures that include, at minimum, parental leave measures for all health workers, in policy or law.
    - Note: Parental leave policies are distinct from maternity or paternity leave. Parental leave refers to long-term leave available to either parent, regardless of gender, allowing them to care for an infant or young child over a period of time. This is typically following an already existing prescribed window of maternity or paternity leave.
      - E.g., in some countries, mothers are able to benefit from a prescribed period of maternity leave, and then extend their leave period by taking advantage of a parental leave benefit.
    - Per ILO Recommendations No. 191 and 165, a period of parental leave should be available to either parent after maternity leave without relinquishing employment or their rights.
  - Partial = The country has social protection measures including parental leave measures available to some, but not all, health workers.
  - No = The country does not have social protection measures including parental leave available to any health worker.
  - ND = Research did not yield any law or policy, i.e., such a policy could exist but could not be found.
- Benchmark
  - Social Protection Mechanisms should be available for all health and care workers, including the provision of health benefits, pregnancy benefits, disability benefits survivor benefits, paid maternity leave, parental leave, childcare leave, unemployment benefits, and older age benefits. Compact at para. 32.
- Database used: ILO Global Care Policy Portal: <https://webapps.ilo.org/globalcare/>. Database was used to verify and code for No. The database mentions a country having the number of weeks of parental leave or having ‘No statutory parental leave’.
- Hierarchy
  - N/A

## I.8 Enabling Work Environments

- Question
  - Does the country have policies/laws for a national system of professional development for health workers that includes: 1) Access to continuing professional development, and 2) In-service training for the health workforce?
- Coding Rule for Primary Source
  - Yes = The country requires both access to continuing professional development and in-service training to be available to health workers.
  - Partial = The country requires either access to continuing professional development or in-service training, but not both, to be available to health workers.
  - No = The country does not require either access to continuing professional development or in-service training to be available to health workers.
  - ND = Research did not yield any law or policy, i.e., such a policy could exist but could not be found.
  - *Sub-Indicators*
    - Sub i: Does the country have national policies/laws for health workers that include access to *continuing professional development*?
      - Yes = Training beyond routine clinical knowledge updates, and includes wide-ranging competencies including research, writing, patient care, professionalism, leadership, *or* management skills are available to health workers by national policy or law.
      - No = None of the enumerated measures above are available to health workers by national policy or law.
      - ND = Research did not yield any law or policy, i.e., such a policy could exist but could not be found.
    - Sub ii: Does the country have national policies/laws for health workers that include *in-service training* for the health workforce?
      - Yes = Training is required to be made available to health workers, and aimed at maintaining and developing competencies necessary for public health needs, under national policy or law.
      - No = No such training is required to be available to health workers by national policy or law.
      - ND = Research did not yield any law or policy, i.e., such a policy could exist but could not be found.
- Coding Rule for NHWA Source
  - Yes = If both sub-indicators are yes
  - Partial = If one sub-indicator is yes, but the other is no.
  - No = If both sub-indicators are no
  - *Sub-Indicators*
    - Sub i: Does the country have national policies/laws for health workers that include access to *continuing professional development*?
      - Yes = If the most recent response on NHWA 1.0 3-08 is “yes” or partial
      - No = If the most recent response on NHWA 1.0 3-08 is “no”
    - Sub ii: Does the country have national policies/laws for health workers that include *in-service training* as an element of national educational plans for the health workforce?
      - Yes = If the most recent response on NHWA 1.0 3-09 is “yes” or partial”
      - No = If the most recent response on NHWA 1.0 3-09 is “no”
- Benchmark
  - Enabling work environments for all health and care workers reflects rights to safe and healthy working conditions and the right to the enjoyment of the highest attainable standard of health. Enabling work environments encompass effective human resource management and training for managers, supportive supervision or feedback, or clear job responsibilities and pathways for progression. Compact at para. 33.
- Hierarchy
  - If there is a conflict between the coding of a primary source, and the results from the NHWA, highlight the conflict. Results from NHWA are voluntarily reported by governments and are not validated by any external source. In most situations, the conclusions drawn from our primary source analysis will be superior to NHWA data. The final decision will be made upon team review.

## I.9 Freedom of Association and Collective Bargaining

- Question
  - Does the country have policies/laws that protect the right to join an independent union or similar workers' organization that apply to health workers?
- Coding Rule for Primary Source
  - Yes = A national policy or law that protects the right to join a union or similar organization that applies to health workers exists, and that right is not subject to government sanction, substantive review, or control and thus is truly independent.
    - Some governments may require a new union or association to register officially with a government agency. Such a requirement may not violate the independence piece of this indicator, so long as that government review is procedural only.
    - *In verifying this indicator, please review additional sources that report on the nature of the government review of the right to freely associate or collectively bargain.*
    - A constitutional provision/protection is **not** sufficient for this indicator to be marked as yes.
  - Partial =Health workers are able to form and join a union, but their right is not truly independent because it is subject to sanction or control by the State.
    - Reports that indicate the right to join a union is not truly independent should be used for verification in addition to the primary source.
  - No = There is no national policy or law that protects the right to join a union or similar organization that applies to health workers.
  - ND = Research did not yield any law or policy, i.e., such a policy could exist but could not be found.
- Benchmark
  - Health and care workers have the right to freedom of association, including the right to form and join trade unions of one’s choice, and the effective recognition of the right to collective bargaining for all health and care workers. Compact at para. 34.
- Hierarchy
  - N/A

## I.10 Whistleblower Protections and Freedom from Retaliation

- Question
  - Does the country have policies/laws providing whistleblower protections that include freedom from retaliation and guaranteed confidentiality to all health workers?
- Coding Rule for Primary Source
  - Yes = National policy or law provides whistleblower protections that include both freedom from retaliation and guaranteed confidentiality. Additionally, all health workers are able to benefit from these protections.
  - Partial = Whistleblower protections may exist, but they include only freedom from retaliation or guaranteed confidentiality, but not both of these provisions and all health workers are able to benefit from these protections.
  - No = National policy or law does not provide for whistleblower protections for all health workers that include either i) freedom of retaliation or ii) guaranteed confidentiality.
  - NA = Research did not yield any law or policy, i.e., such a policy could exist but could not be found.
- Benchmark
  - It is important to protect whistleblowers – those who report and expose malfeasance in the workplace – and all health and care workers from retaliation for seeking to exercise their rights, safeguard their health and safety, and follow their professional ethics. Compact at para. 35.
- Hierarchy
  - N/A
