# Supplementary material for: Laws for health and care worker protection and rights: A study of 182 countries
Source: PLOS Glob Public Health. 2024 Dec 9;4(12):e0003767. doi: 10.1371/journal.pgph.0003767 (PMC11627435; doi:10.1371/journal.pgph.0003767)
Supplement: Attachment — Submitted filename: MEMO Reviewers-HCW Legal-REV 1.2 (submission).docx [file pgph.0003767.s005.docx]

REVIEWER RESPONSE MEMO FOR Kavanagh et al. “Laws for Health and Care Worker Protection and Rights”

Submitted 6 September 2024

| Reviewer Comment | Response | |
| --- | --- | --- |
| **Note to editor:** PLOS has asked us to remove what had previously been tables and make them figures, apologies that this makes things a little complicated. | |  |
| **EDITOR COMMENTS** | |  |
| 1) Transparency in denominators for indicators – the authors note that “Each of the statistics provided in the text below is based on a denominator of the countries with available data.”. As discussed by both reviewers, it would be extremely important to acknowledge missing data by providing the denominator for each indicator in the text and in Figure 3. | It's a good point, we agree and have added denominators in the text so these are transparent as well as the full data in the annex. | |
| 2) Contributions – Further acknowledgement of the multilingual analyst team that conducted the analysis would be important, particularly given the diversity of languages present in the dataset. Reviewer 1 suggests a collaborator author group listing as part of the authors – another option would be a clearer indication of their role in the section on contributors. We would be happy to discuss these or other options for addressing this point. | Thanks very much for this suggestion, we agree that this would be valuable. There are some people who worked on parts of this project bringing language and legal expertise but who would not meet the conditions for authorship under ICJME. We have therefore added a “on behalf of” project line. We include the full list of the team in a new Annex 1, though we would welcome the editors direction if a particular format is preferred. | |
| Please check for consistency between the text and Table 1 (and noted by Reviewer 1). | Thank you, we have edited the text and the tables to more clearly indicate the differences between the locations from which we found primary sources referred to as “national law and policy documents” (e.g. repositories of legal texts) which we analyzed ourselves to create data and the more limited use of secondary sources (e.g. where we use data gathered by others). We see where this generated confusion and have adjusted as such. | |
| Reviewer 2 has also shared a useful suggestion regarding a visualization of the process described on Page 9 (Methodology for Collecting and Evaluating Laws). | Thank you for this suggestion, we have added a new figure (now Figure 1) to illustrate this process. | |
| 4) Table 2 – noted that a graphic designer is working on a revision of this table – if this is now available, please consider submitting with this revision. | Design complete and added here. | |
| 5) Contextual information – Reviewer 2 provides several excellent suggestions on clarifying some key points in the introduction, such as how legal instruments and policy are understood in this analysis. | Agreed. We addressed each of these below and have added text on pg 2-3. | |
| **REVIEWER COMMENTS** | |  |
| Abstract • It would be good to mention in the abstract that the 74th WHA was in 2021 – for readers less familiar with the chronology of WHA. | Good point. We did provide the year, and clarify what exactly happened at that WHA. | |
| Methodology  • The detailed description of each indicator area in the Methodology on pages 4-7 is long in the context of a global public health paper. But on the balance I think it is important to retain. It establishes the rationale for indicator selection and serves additionally as a useful primer for readers seeking background on the significance of each indicator area. | Thank you. We agree it is long we have tried to make it as succinct as we can, including making some changes to make it shorted with this revision, but agree that it is important our indicators, the justification for them, and importantly exactly what we’re measuring and not measuring so readers can evaluate the validity of our measures. | |
| • Relatedly, for indicator 6 (pg 6, para 2/3), including key references in support of salaried CHWs (c.f the work of Svea Closser, and Kenneth Maes) is important. | References Added | |
| • Table 1 is a useful summary, but doesn’t mention some of the sources (e.g. NATLEX database) from the text (or perhaps slightly different nomenclature used?). Please ensure absolute consistency. | Table 1 list 2 distinct types of sources. National law and policy documents, which are collected in multiple ways (e.g. from NATLEX, but the *source* is the national government) and then data for which the *source* is WHO or IRC, which collected information or government surveys.  The suggestion from Reviewer 2 to add a flow-chart, we believe, clarifies this point distinguishing between primary sources and WHO/IRC sources. | |
| • Thank you for the construction of the [www.hcwpolicylab.org](http://www.hcwpolicylab.org) library; this is a huge effort and likely to be an important resource. Given the substantive labour it would be good for the currently listed authors of this paper to include ‘group authorship’ (e.g. the HCW policy lab collaborators) documenting a full list of analysts’ names of those who conducted the individual document analysis. This would acknolwedge the significant linguistic expertise required to enable such analysis across a range of different languages (particularly in Asia) and legal and political contexts. | We agree and have done so, thank you. | |
| Results • One major comment is to recommend the authors more clearly acknowledge and document the proportion of ‘missing data’ for each indicator. Currently (c. pg 10, para 1) the authors note: “for some indicators, no relevant law or policy could be found for some of the countries analyzed or a final determination was not possible. In those cases, we exclude them from our analysis”. However – the authors continue to conduct a comparative analysis of the rates of alignment with each indicator, using summary statistics (%) that imply comparability of the data set for each indicator; when in reality the denominator (total number of countries considered) for each indicator is different. While comparison is still possible it is essential for transparency of this exercise that the different denominator for each indicator is clearly documented at each mention, not just in pie-graph form in Figure 3. So for example on pg 11, para 2 the authors note: “The most aligned area globally is remuneration and fair employment, where 89% of countries have both minimum wage and regulation of working hours for all formally recognized workers.” This sentence does not enable the reader to understand what the 89% refers to. In figure 3, the coverage pie chart shows high level of coverage for that indicator, but the reader must still guess whether the 89% aligned refers to 89% of 150 countries or 160 or 170? It is extremely important to adjust feature of the results whereever reporting on the % alignment of an indicator, to include something like the bold/starred text below: "The most aligned area globally is the remuneration and fair employment, where 89% of countries **for which there was data (X / X)** have both minimum wage and regulation of working hours for all formally recognized workers.”  Adjustments of this sort should be included for the reporting on every indicator in text (noting mentions that need adjusting in the results AND discussion); and the coverage pie charts in Figure 3 should all be amended to include both total numbers in each category and the summary statistic next to the ‘coverage’ title. | The biggest thing we have done to address this throughout the paper is to try again to fill in the missingness. We have not been able to completely address this since some countries’ laws are still not accessible, but we have been able to expand our dataset moderately and the edits reflect these updates.  We have added the proposed denominator notes in the main text, we also note that all data about denominators are available in the annex. | |
| • The challenge of the missing data also adds to some confusion in the opening section of the results (Global Alignment With International Norms…pg 11, para 2, 3). For example, in relation to the statement: 53% of all national laws are fully aligned – what is the denominator here? If the denominator is 178 countries, then presumably full alignment should be adjusted down since the indicator-level assessment reveals missing data in each case. In fact, because of the different denominators (viz number of countries actually able to be evaluated) in each indicator, it isn’t clear to me that this effort to provide a summary analysis across all 10 is appropriate or valid. As it currently stands, Figure 1 inappropriately implies that the proportions of aligned, non-aligned and partially aligned laws applies to all 10 indicators for all 178 countries – when figure 3 shows this can’t be the case. Figure 2 allows more nuance, but again doesn’t reflect the proportion of missing data. Noting here that missing data also tells a story – and transparency in reporting on this is critical to the rigor of this analysis. | We have adjusted the language to clarify the opening section, including the description of Figure 1 (now Figure 2), which we clarified represents a portion of all the laws and policies we analyzed. We have updated the dataset to gain even more coverage (total countries is now 182) and we have removed all countries observations for which we had less than 3 areas covered. The biggest thing we have done to address this throughout the paper is to try again to fill in the missingness. We have not been able to completely address this since some countries’ laws are still not accessible, but we have been able to expand our dataset moderately and the edits reflect these updates. Figure 2 (new figure 5) is now a better reflection using regional %s in order to account for missing data and all of the data throughout have Ns listed to make the missing data more obvious. | |
| • Table 2 while detailed is near impossible to read unless on a large screen with the capacity to zoom in. Some consideration for a format (if at all) appropriate for ‘print publication’ (i.e. the pdf versions) would be good, while retaining the existing version for online use. | We have reformatted Table 2 to make it as readable as possible. | |
| Introduction  - Many readers will probably be not familiar with the Compact, which is the starting point for this study. Could you describe the Compact more in terms of its place in international law. Is it an instrument comparable to CEDAW and/or FCTC? Is it an international commitment that is legally non-binding, just like State commitments to the SDGs? Kindly elaborate further. | Good point, thanks. We have done this – noting that it is not itself legally binding (as, say, CEDAW and FCGH are) – so in the way more akin to the SDGs, but many of its recommendations (e.g., non-discrimination, right to unionize) are in fact binding because they are based on legally binding instruments. | |
| - As many readers of this journal are not lawyers (including myself), I would like the Introduction to include a brief description of the meaning of laws and their hierarchy (e.g., laws vs. circulars vs. guidelines, etc.). This is important because the study examined the legal environment in many countries which included different types of legal instruments and policies. | Well received. Given the vast diversity of the hierarchy of laws in each country, it would be terribly difficult to do this succinctly. For our analysis we focused only on domestic instruments, be it a law, policy, or other measure, that has the force to ensure the human rights standard being assessed. I’ve added a few lines to this effect (pg 3) | |
| - The last paragraph of the Introduction needs an explicit articulation of the research objective. The current statement “we have collected national laws and policies of 178 countries to better understand the legal environment within which health and care workers live and work…” sounds more like a method rather than an objective. What were the research objectives/questions that this study sought to answer? | We have added a more succinct and clear research agenda. (p 4) | |
| Methods - What was the basis for having 178 countries? Please clarify. The WHO has 194 Member States. | We sought to find information for all UN member states and were able to generate data using our methods for 182 out of 194. Given this point, we were actually able to fill in a few more countries in the interim. We have added language to the methods section (p4) to clarify this point.  To address this we have also try again to fill in the missingness. We have not been able to completely address this since some countries’ laws are still not accessible, but we have been able to expand our dataset moderately and the edits reflect these updates. | |
| - The 10 indicators are described without explaining how these were determined. You need to describe the process for identifying them. Was there some form of consultation with stakeholders, review of the literature, or was a workshop held to identify these indicators? | We have added language to Section A of the methodology describing how the indicators were developed. | |
| - The indicators for “ensuring occupational health” namely (a) laws to protect health workers and (b) laws to ensure access to protective equipment/supplies seem inadequate. Occupational health has to do also with ensuring a safe and healthy environment through institutionalised programs in the workplace. | This is a very good point – institutionalized workplace programs are a significant aspect of occupational health. In fact, this is partially captured by our indicator on laws to protect health workers, as the occupational health and safety actions under those laws will likely include these workplace programs. Meanwhile, because of its specificity, as well as the serious weakness in many countries in ensuring health worker access to protective equipment and supplies that was clear during the COVID-19 pandemic, we felt that the indicator on protective equipment/supplies was a good second indicator. Please that these indicators are meant to provide a sense of Compact implementation; they are not intended to be comprehensive in covering all points in the Compact. | |
| - The indicator for “social protection” is the law for parental leaves. This is a very limited indicator. Shouldn’t health insurance coverage for health workers be a measure for social protection? I know firsthand how in some LMICs health workers themselves do not have health insurance coverage (which is an irony). | This is indeed a very limited indicator. Given the social protection is so broad, this was a particularly difficult area for selecting an indicator, as different aspects of social protection are not consistent across countries, and are all quite important. We chose the parental leave indicator for consistency with the National Health Workforce Accounts. We have now added this point into the text and acknowledged that it is limited. | |
| - Table 1 can be improved by grouping the 10 elements according to the four key domains of the Compact. | We consdiered this but upon reflection and discussion with graphic designer, found that doing so created an even busier chart, which was already difficult enough to read. As such, we have respectfully decided to keep it as is. | |
| - I find the description of the methodology for collecting/evaluating laws to be confusing. In systematic/scoping reviews, there is a PRISMA flowchart that provides a summary of the process for article screening. Although this study is not a systematic review and therefore a PRISMA flowchart does not apply, I was wondering if the authors could visualise their process in a diagram, including showing which databases and websites contributed to what, where consultation with networks of civil society came in, how primary and secondary source documents were differentiated, etc. One attribute of good research is reproducibility and without a clear description of the process for finding laws and evaluating them it would be difficult to replicate this study. | We have added a flow-chart as a new Figure 1. | |
| - The HCW Policy Lab (<https://www.hcwpolicylab.org>) looks interesting. Can the authors describe this website more and whether it is related to this project? | We have added text in the methodology B section referencing and describing this website. Thank you. | |
| - I would like to suggest that the codebook is provided as part of the Annex. | We have done so thank you. Noting that most of the codebook is included in table in the paper. | |
| - I cannot find it in the text of the Methods – what is the criteria for classifying laws as aligned, partially aligned, or non aligned? I think I saw the criteria somewhere in the Annex but this information should be in the Methods. | The criteria for aligned, partial, and not aligned are explicitly outlined in Table 1 for each indicator and marked as such. We added to the column title to make this more obvious. | |
| - “Countries where research did not yield any law or policy answers were classified as no data—reflecting that such a measure could exist but could not be found.” – What about the possibility that there was in fact no such law in the country? | We have amended the text to clarify that this is indeed a possibility. | |
| - And how did you account for laws that were written in the non-Latin alphabet (e.g., laws in Chinese characters and thus may not be searchable online), and laws that may only exist offline (i.e. only in print)? | Great question! Where possible, we conducted the search for laws in the national script of the target country (e.g. someone with fluency in Arabic would search with an Arabic keyboard). For countries where this was not possible, translation software was used.  We tried to access laws that may exist only offline by relying on our networks to share copies where possible. Sometimes this would be a scan or a photo of the relevant text. | |
| Results - I think Figure 1 can be excluded as describing the alignment of 1,238 laws as a whole without unpacking the countries is not really policy useful. The information can simply be presented in the text without a need for Figure 1. | Here we respectfully disagree. Our objective is to measure alignment of national laws with the Global Compact to better understand the legal environment within which health and care workers live and work. At the highest level, this measure of overall global alignment of national laws with the global compact gives a sentinel measure for international policy makers to consider. A fully aligned world would be a fully blue pie graph—this shows the distance to that goal agreed by member states. To us it is a key piece of evidence for our note in the discussion that implementing the Compact “will require extensive and continuous reform.” As such, we propose to retain this figure (now figure 2).  However, to simplify we have removed what was previous figure 2 *Countries' Alignment of Health and Care Workers' Laws and Policies with Global Norms* following our further thinking on the reviewers’ feedback. | |
| - Figure 2 – please place the numbers of countries on the bars in addition to the percentages. | Figure 2 has been replaced with a new figure (current figure 5) which reflects percentage by region reather than absolute numbers, reflecting reviewer comments about shifting N. | |
| - For the purpose of regional action, I think it would be useful to unpack 4 according to the WHO regions. Action can be taken at the regional levels to address some of the gaps. For example, showing how WPRO compares to SEARO etc. would be a useful analysis, including which countries within each region aligned well (or did not align well) with the Compact. | We agree and we have removed figure 2 and replaced it with a regional graph, which is now new figure 5. | |
| - Figure 3 – can you put the numbers of countries on the bars and the pie charts? | While we appreciate the reviewers’ goal here, when we tried to add more numbers to the figures they became even less readable. So instead we have included the number of countries in the text in addition to the detailed breakdown that is retained in the annexes as in the original submission. With this addition, we believe we have accomplished the important goal of making the “coverage” transparent. | |
| And why are there no partially aligned countries for some indicators (e.g. 3, 6.1/6.2, 8.1/8.2)? | Partial alignment is available for all indicators for which a meaningful part-way could be distinguished for an indicator—which was not true for all indicators. This is described in the detailed discussion in Methodology section A, but to signpost this we have added text to the methodology section making this clearer. | |
| - I think you should also mention and discuss the indicator with the least data (10. whitleblower protections) and most data (6.3 remuneration). | We agree and have added this at the end of the results section. | |
| - Table 2 – rather than have the countries in alphabetical order, I think it would be more useful if they are clustered according to WHO regions within the same Table. | We explored this with the graphic designer, who felt already busy graph became too cluttered when regional sections were added. The WHO regions are also not always intuitive to others so in the end we kept it alphabetical, but thank the reviewer for their idea. | |
| - In the last paragraph of Results, there are statistical findings related to country income and country region which seemed out of place as such analyses were not part of the Methods. If you will include these Results here, then the statistical analysis should have been described in the Methods. I saw some of the computations in the Annex but the statistical approach has to be described in the Methods. | Added text to the methods section on this point. Thank you. | |
| - I do not like the word “idiosyncratic” to describe lawmaking in the last paragraph. Can the authors please reformulate. | Removed. | |
| Discussion  - “Only one country is coded as fully aligned with all 10 indicators.” – Which country was that? This should have been mentioned in the Results. Likewise, was there a country that was least aligned? | Thank you. We have broken out the answers to this question and clarified after some re-analysis and included example countries in this and other sessions. | |
| - “Notably, 64 countries are aligned with half or more of the indicators studied.” – Is there anything common about these 64 that might explain why they were aligned with the Compact? | This is a good question we hope to explore. For this paper, we conducted some initial analysis by geography and income to test two common theories that might explain alignment—and while we report a weak relationship between income category and alignment we are not confident this is meaningful; geography was not a significant predictor of alignment. We hope to do significant further work on this question including qualitative work that we think will likely be necessary and some more advanced quantitative work, but doing so is outside the scope of this paper which is largely descriptive. | |
| - Since the starting point of this study was the Compact, I wonder if the authors could also reflect about the role of the Compact in advancing more legislation globally to cover more of the elements of the Compact, including some reflections on State compliance and accountability. The first author of this study had a fine article in the Lancet about State compliance to international commitments which could inform this part of the Discussion. | This is a very good point and helpful suggestion.  We have rewritten the conclusion to do so. | |
| Conclusion  - The last few sentences of the Conclusion seem anticlimactic and refer to ideas for future research that should be combined instead with the future research section of the Discussion. | Agreed. Conclusion is rewritten and expanded. | |
| Minor comments  - Reference numbers 10, 25, 36, 38, are incomplete. | Thank you these are fixed. | |
| - WHO is the funder of this study as well as part of the authorship. A COI should be declared. | Will seek editor advice on how best to approach this. | |
| - For the revised manuscript, please put line numbers at the margins so it would be easier to refer to the text next time. | Done | |
| Abstract  - Please revise the Abstract in light of the changes to be undertaken above. | We have done so. | |
