# Supplementary material for: Laws for health and care worker protection and rights: A study of 182 countries
Source: PLOS Glob Public Health. 2024 Dec 9;4(12):e0003767. doi: 10.1371/journal.pgph.0003767 (PMC11627435; doi:10.1371/journal.pgph.0003767)
Supplement: Attachment — Submitted filename: MEMO Response to Reviewers-PLOSGPH HCW Paper REV 2.docx [file pgph.0003767.s006.docx]

REVIEWER RESPONSE MEMO #2 FOR Kavanagh et al. “Laws for Health and Care Worker Protection and Rights”

Submitted 24 October 2024

| Reviewer Comment | Response | |
| --- | --- | --- |
| **EDITOR COMMENTS** | |  |
| Thank you for the comprehensive efforts in this revision. We are recommending a minor revision based on the reviewer's comments regarding the proportion of countries included in the analysis in the text and - if possible - in Figure 3. I note the comment in your response regarding the difficulty of including numbers within the figure, but one option is to include the denominator under the pie chart on coverage. This will help with the interpretation of the corresponding bar charts. Happy to discuss this further to identify a solution. | Thank you, as agreed via email we have done as agreed:  -Removing the pie-charts  -Instead including the denominator for each indicator under the corresponding bar chart with (n=YYY) as the label. | |
| **Review Comments to the Author** |  | |
| * While the authors have added the denominators to the text discussing coverage these remain in an ambiguous format. It is not immediately clear that the (n=xxx) number is in fact a denominator. To make this clearer, it would be very helpful if the format suggested in the original comments were used (see below). This ensures absolute clarity for the reader.  EG: CURRENT: "Just 1% of countries are not aligned (n=172)" RECOMMENDED FORMAT: "Just 1% (2/172) of countries are not aligned." [NB: retro engineered the numerator here based on the figures for this example].  * Second, regarding Figure 3, as both reviewers mentioned in the first round it is important to ensure the addition of the denominators to ensure transparency of coverage. Accurate representation of data is as important a consideration as readability, and it its current state Figure 3 still gives a potentially false impression that the coverage of each group is based on an identical number of countries. While the authors have now included this information in the text which is great - it is important for (any) figure to contain all information necessary for a reader to fully understand what is being represented, independent of accompanying text or an appendix. Either by using foot notes, or by adding a x/x figure next to each pie chart, or by other means, this information is critical to the message conveyed by this figure. | *We note this suggestion and have done as requested. Instead of including the denominator at the end of the paragraph we have moved it to the start of the paragraph.  The text now reads, for example, “While over two thirds (69%, 94/137) of countries researched have some form of health services for all health workers…” (p11)  *As agreed with editor, we have done as the reviewer requests. Since we risked being repetitive with the same information in the figure twice (and in the text and the annex), we have removed the pie charts which the reviewer found confusing and instead have placed the denominator for each indicator under the corresponding graph. | |
